# Electronic Health Record–Driven Approaches in Primary Care to Strengthen Hypertension Management Among Racial and Ethnic Minoritized Groups in the United States: Systematic Review

**DOI:** 10.2196/42409

**Published:** 2023-09-15

**Authors:** Dominik Ose, Emmanuel Adediran, Robert Owens, Elena Gardner, Matthew Mervis, Cindy Turner, Emily Carlson, Danielle Forbes, Caitlyn Lydia Jasumback, John Stuligross, Susan Pohl, Bernadette Kiraly

**Affiliations:** 1 Department of Family and Preventive Medicine University of Utah Salt Lake City, UT United States; 2 Community Physicians Group University of Utah Salt Lake City, UT United States; 3 Utah Department of Health and Human Services Salt Lake City, UT United States

**Keywords:** hypertension, electronic health record (EHR), health data, EHR-driven, primary care, racial and ethnic minority groups

## Abstract

**Background:**

Managing hypertension in racial and ethnic minoritized groups (eg, African American/Black patients) in primary care is highly relevant. However, evidence on whether or how electronic health record (EHR)–driven approaches in primary care can help improve hypertension management for patients of racial and ethnic minoritized groups in the United States remains scarce.

**Objective:**

This review aims to examine the role of the EHR in supporting interventions in primary care to strengthen the hypertension management of racial and ethnic minoritized groups in the United States.

**Methods:**

A search strategy based on the PICO (Population, Intervention, Comparison, and Outcome) guidelines was utilized to query and identify peer-reviewed articles on the Web of Science and PubMed databases. The search strategy was based on terms related to racial and ethnic minoritized groups, hypertension, primary care, and EHR-driven interventions. Articles were excluded if the focus was not hypertension management in racial and ethnic minoritized groups or if there was no mention of health record data utilization.

**Results:**

A total of 29 articles were included in this review. Regarding populations, Black/African American patients represented the largest population (26/29, 90%) followed by Hispanic/Latino (18/29, 62%), Asian American (7/29, 24%), and American Indian/Alaskan Native (2/29, 7%) patients. No study included patients who identified as Native Hawaiian/Pacific Islander. The EHR was used to identify patients (25/29, 86%), drive the intervention (21/29, 72%), and monitor results and outcomes (7/29, 59%). Most often, EHR-driven approaches were used for health coaching interventions, disease management programs, clinical decision support (CDS) systems, and best practice alerts (BPAs). Regarding outcomes, out of 8 EHR-driven health coaching interventions, only 3 (38%) reported significant results. In contrast, all the included studies related to CDS and BPA applications reported some significant results with respect to improving hypertension management.

**Conclusions:**

This review identified several use cases for the integration of the EHR in supporting primary care interventions to strengthen hypertension management in racial and ethnic minoritized patients in the United States. Some clinical-based interventions implementing CDS and BPA applications showed promising results. However, more research is needed on community-based interventions, particularly those focusing on patients who are Asian American, American Indian/Alaskan Native, and Native Hawaiian/Pacific Islander. The developed taxonomy comprising “identifying patients,” “driving intervention,” and “monitoring results” to classify EHR-driven approaches can be a helpful tool to facilitate this.

## Introduction

Worldwide, and in the United States, hypertension is one of the most common chronic conditions. It is estimated that 1.28 billion globally and 116 million people in the United States alone live with hypertension, with far-reaching consequences [1,2]. For example, in 2019, hypertension contributed to or caused more than 500,000 deaths in the United States [3]. In addition, hypertension is a significant contributor to cardiovascular disease morbidity and mortality [4] and has been linked to sudden cardiac arrest and death [4,5]. Each year, hypertension accounts for about US $131 billion to $198 billion in health care services, medications, and loss of productivity from premature death [6].

The burden of hypertension is much higher in underrepresented ethnic and minoritized groups, including those who are Black/African American, American Indian/Alaskan Native, American Asian, and Native Hawaiian Pacific Islander [7-9]. In particular, Black/African American people have the highest prevalence of hypertension morbidity and mortality in the United States [10]. Overall, the total mortality contribution for Black/African American adults equates to 3.8 million potential years of life lost, about 30% to 60% greater years of life lost compared to White adults [11]. Concerning the Hispanic/Latino population, every fifth participant in the Hispanic Community Health Study developed hypertension during the 6-year study period. The incident rates for hypertension among Hispanic/Latino people of Caribbean background were substantially higher [12]. Due to disparities in health literacy, access, and education, the prevalence of hypertension is expected to increase in the Hispanic/Latino population [13].

There is an ongoing debate on how health care systems can better meet the needs of racial and ethnic minoritized groups [14]. Primary care has emerged as a potentially viable means of improving overall health outcomes in these populations [15,16]. Often regarded as the first point of entry into the health system, primary care has shown promise in the efforts to reduce health disparities [17]. Data-driven approaches based on electronic health records (EHRs) may be an effective approach for improving cardiovascular health outcomes, including hypertension, in racial and ethnic minoritized groups [18,19].

However, evidence for using EHR in data-driven approaches in primary care to improve hypertension management in these populations in the United States remains scarce. This review aims to bridge this gap.

## Methods

### Protocol

We utilized the PICO (Population, Intervention, Comparison, and Outcome) process (Table 1) to develop the database search strategy, inclusion and exclusion criteria, and protocol for this systematic review. The comparison element in the guideline was replaced with “setting” since we did not focus on evaluating or comparing interventions. We used the PRISMA (Preferred Reporting Items for Systematic Reviews and Meta-analyses) guidelines [20] for conducting this review (Multimedia Appendix 1). The study protocol was not registered.

**Table 1:** Study inclusion and exclusion criteria.

PICO^a^ element and inclusion criteria				Exclusion criteria
**Population**				
	**Racial and ethnic minoritized groups in the United States**			
		Asian; African American/Black; Hispanic/Latino; Hawaiian; Pacific Islander; American Indian; Alaska Native; minority; underserved; disadvantaged; priority population	Study not conducted in the United States; no racial or ethnic minority listed or described	
**Intervention**				
	**EHR^b^-driven**			
		Patient EHR; EMR^c^; CDS^d^; dashboard; eHealth; routine data; clinical data	No mention of data source or how data were used	
**Outcome**				
	**Primary outcome**			
		Hypertension management; BP^e^ management	Outcome of interest not related to hypertension or BP management	

^a^PICO: Population, Intervention, Comparison, and Outcome.

^b^EHR: electronic health record.

^c^EMR: electronic medical record.

^d^CDS: clinical decision support.

^e^BP^:^ blood pressure.

### Search Strategy

A comprehensive search of eligible studies was conducted via Web of Science and PubMed. The first group of searches was performed in September 2021. The database search was undertaken using the keywords described in Table 2. We restricted our literature searches to English-language publications and United States–based studies. In addition to the systematic search, we used a snowball procedure to identify additional studies by searching the reference lists of the included publications.

**Table 2:** Search query.

Search field	PICO^a^ category	Web of Science query
1	Outcome	“Hypertension” OR “high blood pressure” OR “cardiovascular disease” OR “heart disease”
2	Population	Asian* OR Black* OR “African American” OR “Hawaiian” OR “Pacific Islander” OR “American Indian” OR “Alaska Native” OR “Hispanic” OR “Latino” OR “minority” OR “priority population” OR “disadvantaged population“ OR “underserved population”
3	Setting	“primary care” OR “family medicine” OR “outpatient care” OR “preventive medicine” OR “primary health care” OR “community health”
4	Intervention	“Dashboard” OR “data-driven” OR “data visualization” OR “electronic health” OR “eHealth” OR “electronic health record” OR “routine data” OR “clinical data” OR “digital data”

^a^PICO: Population, Intervention, Comparison, and Outcome.

### Selection Process

First, 2 authors (EA and DO) identified potentially eligible articles by screening the titles and abstracts. The articles were assessed again through a full-text review to determine whether they fit with the study aims. The researchers discussed the findings. Inconsistencies and discrepancies were addressed until a consensus was obtained and an agreed-upon solution was applied. A summary of the inclusion and exclusion criteria is provided in Table 1.

### Data Collection Process

An initial independent review of all the included articles was conducted by 3 authors (EA, CT, and DO). Then, in pairs, comparisons were made. Disagreements were discussed until a consensus was reached.

All articles of interest were marked up in the databases and imported directly into Zotero citation management software (version 5.0.96.3; Corporation for Digital Scholarship). The articles were grouped in Zotero by title and abstract, intervention type, and selection status. Relevant extracted data, including author, year of publication, data utility, study focus, results, and intervention type, were entered into a summary table created in Microsoft Word (Microsoft Corp) and reviewed independently.

### Data Items and Synthesis Methods

For all searches, we extracted information relating to the characteristics of the included studies. The characteristics consisted of author and publication year, study population, study aims, utilization and role of the EHR, and the intervention setting. For the study population, we reported the race and ethnicity of the participants (Table 3).

**Table 3:** Synthesis methods.

Synthesis methods	Description
Primary characteristics	Author name, study title, and publication year
Study population	Descriptive analysis of all racial and ethnic minoritized groups receiving intervention
Aim/approach	Overall outcome of intervention and intervention implemented to achieve the outcome
Intervention setting	Primary location in which the intervention was implemented
EHR^a^-driven aspect	Role of the EHR

^a^EHR: electronic health record.

### Risk of Bias Assessment

A bias assessment was conducted using the Cochrane Risk-of-Bias version 2 (RoB 2) tool for randomized controlled trials (RCTs), the RoB 2 cluster tool for cluster randomized trials (CRTs), and the Risk of Bias in Nonrandomized Studies (ROBINS-I) tool by 3 authors (EA, MM, and DO). All 3 bias tools were assessed using Microsoft Excel and individually uploaded to the *robvis* package for synthesis [21].

## Results

### Study Selection

**Figure 1:**
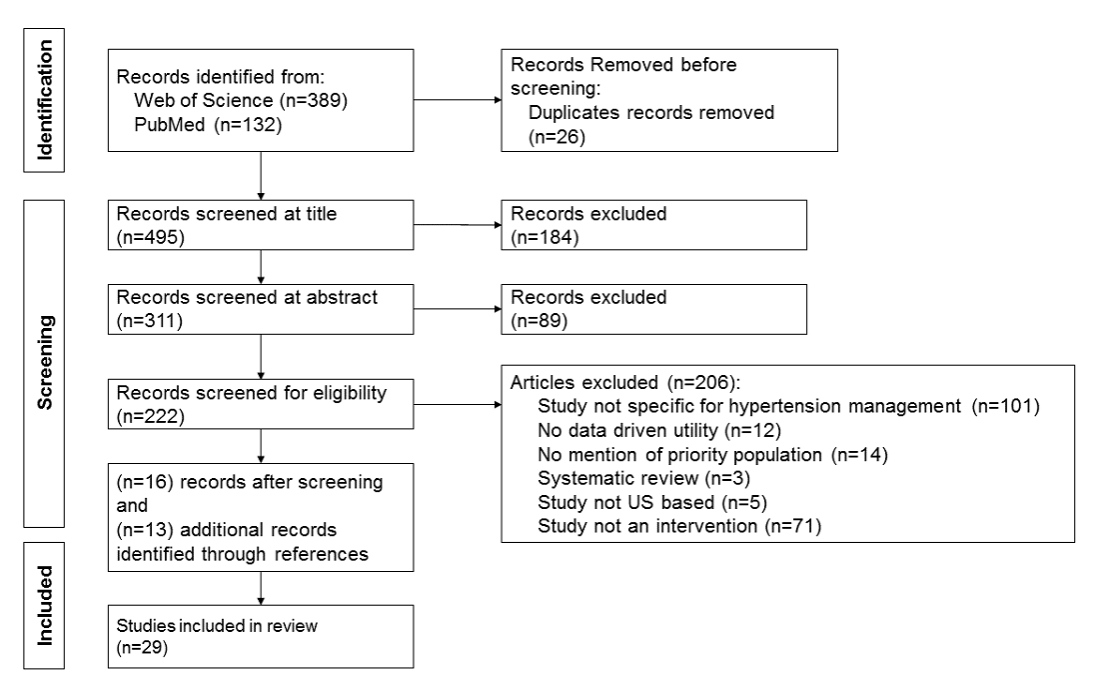
Study flowchart.

We identified a total of 521 publications from the Web of Science (n=389,75%) and PubMed (n=132, 25% databases (Figure 1). We removed 26 duplicates, leaving 495 articles for screening. After the title screening for hypertension, data, or racial and ethnic minority–related terms, we excluded 184 publications. The remaining 311 publications were screened at the abstract level, and 89 publications were excluded as they did not mention hypertension or racial and ethnic minoritized groups. Of the remaining 222 articles, 205 were excluded for the following reasons: the study was not specific to hypertension management, data-driven utility was not available, there was no mention of a racial and ethnic minoritized group, the study was a systematic review, it was not based in the United States, and the study was not an intervention. We added 13 more records using the reference lists of included studies. This resulted in 29 studies that met all the inclusion criteria. We did not use any automation tools in the study selection process. All records were manually screened by 3 authors (EA, CT, and DO).

### Study Characteristics

We categorized all included articles based on observed differences in the study design, study populations, approach, primary and secondary outcomes of interest, duration, and the role of EHR. We first present the results of the risk of bias assessments. Then, we describe the findings for each of the observed characteristics for study design and population. Next, we report findings on the same characteristics for primary aims, approach, and role of EHR, duration, and results.

### Risk of Bias Assessment

We performed assessments for studies that were RCTs (Multimedia Appendix 2), CRTs (Multimedia Appendix 3), and non-RCTs (Multimedia Appendix 4). Overall, 18 (62%) studies were judged to be at low risk of bias [22-39], 1 (3%) study was judged to be high risk [40], and 10 (34%) studies had some concerns [13,41-49]. Of these, 6 (60%) studies had some concerns relating to the randomization process [40-44,46] and another (n=1, 10%) due to confounding [47]. In addition, 2 (20%) studies had some concerns regarding the outcome assessors and participants’ awareness of the interventions but likely did not influence the outcome [13,47]. Another study (n=1, 10%) had some concerns about handling missing data [48]. Moreover, 5 (50%) studies had some concerns regarding the selection of reported results due to the lack of information about a prespecified analysis protocol [30,41,43,45,46].

### Study Design

A total of 18 (62%) studies were RCTs [13,22-32,40-44,49] (Table 4). Other study designs included 3 (10%) CRTs [34,46,46] and 8 (28%) non-RCTs [34-39,47,48]. The non-RCTs included 2 (25%) pragmatic studies [35,36], 3 (38%) retrospective cohorts [38,39,48], 1 (13%) quasi-experimental study [37], 1 (13%) feasibility study [34], and 1 (13%) quality improvement study [47]. Table 4 includes an overview of the included studies and their populations**.**

**Table 4:** Overview of the included studies and their populations.

Study characteristics				Overall studies (N=29), n (%)		Overall patients (N=73,039), n (%)		Black/African American patients (n=52,666), n (%)		Asian American patients (n=6527), n (%)		American Indian/Alaskan Native patients (n=96), n (%)		Hispanic/Latino patients (n=13,750), n (%)
**Setting**														
	Clinical-based			15 (51.74)		28,369 (38.84)		12,887 (45.42)		5994 (21.12)		—^a^		9488 (33.44)
	Home-based			13 (44.8)		44,230 (60.56)		39,779 (89.93)		93 (1.42)		96 (0.22)		4262 (9.64)
	Community-based			1 (3.44)		440 (0.60)		—		440 (100)		—		—
**Design**														
	RCT^b^			18 (62.06)		5647 (7.31)		3726 (65.98)		551 (9.76)		96 (100)		1274 (22.56)
	CRT^c^			3 (10.34)		6253 (8.56)		6044 (96.66)		128 (2.04)		—		81 (1.43)
	**Non-RCT**			8 (27.58)		61,020 (83.54)		42,896 (70.29)		5848 (9.58)		—		12,276 (20.12)
		Quality improvement	1 (12.50)		40,808 (66.88)		37,359 (91.54)		—		—		3449 (8.45)	
		Pragmatic	2 (25)		12,734 (20.86)		3696 (29.02)		5506 (43.24)		—		3532 (27.74)	
		Retrospective	3 (45)		6969 (11.42)		1728 (24.80)		—		—		5241 (77.79)	
		Quasi-experimental	1 (12.50)		506 (0.82)		110 (21.73)		342 (67.59)		—		54 (10.67)	
		Feasibility	1 (12.50)		3 (0)		3 (100)		—		0 (0)		0 (0)	
**Intervention**														
	Health coaching			8 (27.58)		2024 (2.77)		1171 (57.86)		102 (5.03)		74 (3.66)		677 (33.44)
	BPA^d^			3 (10.34)		43,489 (59.54)		38,973 (89.62)		342 (0.78)		—		4174 (9.60)
	DMP^e^			3 (10.34)		13,315 (18.22)		4127 (31)		5506 (41.35)		—		3682 (27.65)
	Telemedicine/virtual visits			3 (10.34)		674 (0.92)		635 (94.21)		—		22 (3.26)		17 (2.52)
	Home BP^f^ monitoring			3 (10.34)		810 (1.11)		252 (31.11)		8 (0.99)		—		549 (67.78)
	CCM^g^			2 (6.89)		5575 (7.63)		1039 (18.64)		—		—		4536 (81.36)
	CDS^h^			1 (3.44)		440 (0.60)		231 (52.50)		128 (29.09)		—		81 (18.41)
	Medication management			2 (6.89)		950 (1.30)		916 (96.42)		—		—		34 (3.58)
	Self-management			2 (6.89)		752 (1.02)		312 (41.48)		440 (58.51)		—		—
	Dashboard			1 (3.44)		4774 (6.54)		4774 (100)		—		—		—
	Case management			1 (3.44)		236 (0.32)		236 (100)		—		—		—
**Role of EHR^i^**														
	Identification			25 (86.20)		71,782 (33.49)		51,981 (72.42)		6074 (8.46)		96 (0.13)		13,743 (19.14)
	Intervention			21 (72.41)		72,085 (33.64)		47,419 (65.78)		6509 (9.02)		96 (0.13)		18,061 (25.06)
	Monitoring			17 (58.62)		70,408 (32.86)		47,289 (67.16)		5941 (8.44)		22 (0.03)		17,156 (24.36)

^a^—: not applicable.

^b^RCT: randomized controlled trial.

^c^CRT: cluster randomized trial.

^d^BPA: best practice alert.

^e^DMP: disease management program.

^f^BP: blood pressure.

^g^CCM: chronic care model.

^h^CDS: clinical decision support.

^i^EHR: electronic health record.

### Study Populations

All the included studies were conducted in the United States. As shown in Table 4 and Multimedia Appendices 5-7, studies in our review differed in the inclusion of racial and ethnic minoritized groups. Black/African American participants (n=52,666, 72.11%) were the largest population studied, followed by Hispanic/Latino (n=13,750, 18.82%) and Asian American (n=6527, 8.94%) participants. Of the studies reviewed, Bolen et al (2021) [47], Schroeder et al (2020) [25], Fontil et al (2018) [36], and Patel et al (2018) [33] had the largest and most diverse cohort, which included participants who were Asian, Hispanic, American Indian/Alaskan Native, and Black/African American. A total of 5 (17%) studies focused on a single racial and ethnic minority cohort [13,24,28,31,46]. The cohorts of studies by Ogedegbe et al (2014) [46], Pezzin et al (2011) [24], and Artinian et al (2007) [28] consisted of Black/African American patients. Meanwhile, Kim et al (2014) [31] focused on Korean American patients, and Schoenthaler et al (2020) [13] studied Hispanic/Latino patients.

### Study Settings

We identified 3 study settings in our review: clinical, home, and community based. We defined clinical-based as studies in which participants received the intervention in a primary care clinic, home-based as studies in which participants received the intervention at home, and community-based as studies in which participants received interventions involving community resources or interventions conducted in a community setting.

Most studies (n=14, 48%) in our review were clinical-based [13,26,27,34-41,45,46,48]. Only 1 (7%) study enrolled patients who received care from a health care center and who were also assigned to a primary care provider [34]. In addition, there were 13 (45%) home-based studies [22-25,28-30,32,42-44,47,49]. Among those, the study by Pezzin et al (2011) [24] assigned participants to a nurse-led, home-based care intervention. Finally, we identified 1 (3%) community-based study. This study by Kim et al (2014) [31] recruited and assigned patients based on a geographically defined community area. These areas were within service reach of a rural health care center.

### Primary Aims

A common primary aim across the included studies was to improve overall blood pressure (BP) control [22,24,29,31,36,37,39,42-44,46-48]. A total of 5 (17%) studies aimed to reduce both systolic BP (SBP) and diastolic BP (DBP) [26,30,32,38,41], and an additional 3 (10%) studies aimed to reduce overall BP [25,27,28]. One (3%) of the studies had more than 1 primary study aim. To illustrate, the study by Schroeder et al (2020) [25] aimed to reduce both overall BP and improve medication adherence. Other studies aimed to reduce overall SBP [23,35], improve medication adherence [13,49], increase the diagnosis of elevated BP [45], improve self-management [34,40], and increase guidelines-adherent statin prescriptions [33].

### Interventions

The most common EHR-integrated approach to strengthen hypertension management was a health coaching intervention (n=8, 28%). For example, the study by Persell et al (2020) [23] used a smartphone–based health coaching system to reduce SBP and increase patients’ self-confidence in reducing their BP. Their smartphone coaching app was powered by an artificial intelligent system to reduce BP and promote self-management of hypertension. Other common EHR-driven approaches were related to disease management programs (DMPs) [26,36,41], best practice alerts (BPAs) [37,47,48], telemedicine [28,43,44], web-based interventions [29,32], chronic care models (CCMs) [39,46], medication management [27,38], and self-management [30,31]. To illustrate, Jackson et al (2012) [41] implemented a hypertension DMP to reduce both SBP and DBP, while Artinian et al (2007) [28] implemented a telemedicine intervention to reduce BP in Black/African American study participants.

### Role of the EHR

**Figure 2:**
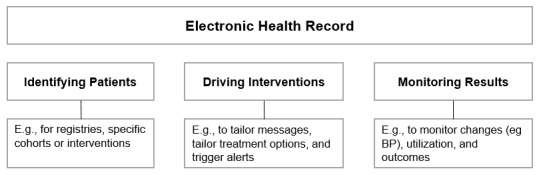
Taxonomy to classify electronic health record (EHR)–driven approaches. BP: blood pressure.

All the included studies used the EHR to identify patients, drive interventions, or monitor results. We define “identifying patients” as the use of the EHR to identify specific patient groups or populations. “Driving intervention” refers to the use of the EHR in the implementation of a study intervention. Finally, “monitoring results” refers to any use of the EHR to assess or evaluate outcomes (Figure 2, Table 5).

With respect to “identifying patients,” 25 (86%) of the included studies mentioned using the EHR in some way to identify eligible patients. Additionally, 4 (16%) studies built a health registry based on information provided by the EHR [25,36,37,47]. Lopez et al (2019) [37] used the EHR to build a patient hypertension registry report. This registry was then used to identify patients with a diagnosis of hypertension and uncontrolled BP at their most recent clinic visit and to create a list of patients for follow-up. Other studies retrieved patient information directly from the EHR.

Regarding “driving interventions,” 21 (72%) studies mentioned using the EHR as part of their interventions. The EHR was used to either trigger or alert processes and actions [27,33,36,37,41,45,47,48], to alter the course of treatment or intervention [13,22,23,25,29,31,32,38,39,42,43], or both [33,41]. To illustrate, Schroeder et al (2020) [25] integrated the EHR with an interactive text messaging system to send culturally tailored motivational messages. In another study, existing EHR tools were used to drive the primary intervention. Fontil et al (2018) [36] utilized the EHR to create an internal hypertension registry that helped facilitate patient outreach and feedback for provider performance during the intervention period. Additionally, Lopez et al [37] used the EHR to trigger medical alerts. These alerts contained remainders for providers to ensure follow-up appointments were being scheduled based on patients’ BP control status. The EHR was also used to send out and document order sets, including lab tests and prescriptions.

Finally, 17 (59%) studies mentioned using the EHR for “monitoring results” [13,22,23,29,30,32-34,36-39,41-43,47]. For example, Tilton et al [38] used the EHR to track reduction in SBP and DBP and clinic visits, and Schoenthaler et al (2020) [13] linked patients’ EHRs to an electronic monitoring device to assess changes in BP and medication adherence. Persell et al (2020) [23] used EHR data to report the frequency of telephone, office, and data portal usage during the study period.

**Table 5:** Role of the EHR^a^.

Use case		Included studies	Studies, n (%)
**Identifying patients**			
	Identify the eligible patients	All except Artinian et al [28], Bove et al [43], Kim et al [31], and Persell et al [23]	25 (86)
**Driving interventions**			
	Tailored messages (behavioral, educational, coaching)	Bove et al [43], Green et al, [32] Kim et al [31], Persell et al [23], Schoenthaler et al [13], and Schroeder et al [25]	6 (21)
	Tailored medication and/or treatment plans	Jackson et al [41], Margolius et al [22], Margolis et al [44], Magid et al [29], Yi et al [42], Turner et al [39], Persell et al [27], and Tilton et al [38]	8 (28)
	Trigger alerts (related to patient, BP^b^/medication, best practices)	Jackson et al [41], Kharbanda et al [45], Persell et al [27], Patel et al [33], Lopez et al [37], and Swedlund et al [48]	6 (21)
	Trigger benchmark targets	Fontil et al [36] and Bolen et al [47]	2 (7)
	Collect/transmit BP readings	Artinian et al [28], Magid et al [29], and Kim et al [31]	3 (10)
**Monitoring results**			
	Monitor BP changes	Jackson et al [41], Bove et al [43], Margolis et al [44], Magid et al [29], Green et al [32], Yi et al [42], Fontil et al [36], Tilton et al [38], Schoenthaler et al [13], and Bolen et al [47]	10 (34)
	Prescriptions and medications (incl. changes/adherence)	Margolius et al [22], Turner et al [39], Patel et al [33], Lopez et al [37], and Schoenthaler et al [13]	5 (17)
	Encounters/visits (primary care, emergency department, hospital)	Margolius et al [22], Yi et al, [42] Lopez et al [37], Tilton et al [38], and Persell et al [23]	5 (17)
	Other (communication, lab results, adverse events)	Bosworth et al [30], Johnson et al [34], and Lopez et al [37]	3 (10)

^a^EHR: electronic health record.

^b^BP: blood pressure.

### Study Duration

The studies included in our review varied in their intervention duration. Most studies (n=7, 24%) were conducted over a 6-month [13,22,23,29,32,35,43] or 12-month (n=6, 21%) [25,28,37,40,45,46] period. Other studies reported a duration of 3 months (n=2, 7%) [24,34], 24 months (n=3, 10%) [30,39,49], 2 months (n=1, 3%) [33], 11 months (n=1, 3%) [48], 9 months (n=1, 3%) [42], and 5 years (n=1, 3%) [47]. Additionally, 7 (24%) studies had multiple time intervals. Of these, 3 (43%) studies reported intervals of 6, 12, and 18 months [22,41,50]. The remaining 4 (57%) studies reported intervals of 3,6, and 12 months [27]; 6 and 12 months [38]; 9 and 18 months [26]; and 15 and 24 months [36].

### Outcomes and Impact

We observed mixed results relating to the impacts of these interventions on strengthening hypertension management. Of the 29 studies included in the review, 22 (76%) reported a significant change in their respective outcomes. These results differed by study design, aims, population, approach, setting, role of EHR, and study duration.

With respect to study aims and population, 8 (62%) studies with the primary aim of improving overall BP control reported a statistically significant change in BP [22,24,29,36,39,47-49]. In addition, the racial and ethnic minoritized groups in these studies were predominantly Hispanic/Latino and Black/African American. Another 10 (34%) studies reporting a significant improvement in one or more of their outcomes included studies with the primary aim of reducing SBP and DBP [13,26,38,41,44], improving self-management of BP monitoring [34], increasing the diagnosis of elevated BP [45], increasing rates of statin prescriptions [33], increasing self-confidence in BP control [23], and following treatment protocols [49].

For study design, 7 (88%) out of 8 non-RCTs [34,36-39,47,48] and 2 (67%) out of 3 CRTs [33,45] reported a significant change in their aims and outcomes. Among the non-RCTs, there was the feasibility study by Johnson et al (2016) [34], the quality improvement study by Bolen et al (2021) [47], the quasi-experimental study by Lopez et al [37], the pragmatic study by Fontil et al (2018)[36], and the retrospective studies of Swedlund et al (2019) [48], Tilton et al (2019) [38], and Turner et al (2018) [39].

In terms of study setting, the majority of studies that reported a significant change in at least 1 of the intended outcomes were clinical-based studies (n=10, 34%) [13,33,34,36-39,41,45,48]. Among all the included clinical-based studies, 2 (20%) had results that differed in BP outcomes. Both Hebert et al (2012) [26] and Schoenthaler et al (2020) [13] showed a significant reduction in SBP but not in DBP. Next, 7 (54%) home-based studies reported a significant change in their outcomes [22,24,29,30,44,47,49]. Among all the included home-based studies, 2 (29%) studies reported results that differed by aim [23,28]. The study by Artinian et al (2007) [28] led to a significant reduction in SBP but no significant reduction in DBP. Meanwhile, Persell et al (2020) [23] reported a significant increase in patients’ self-confidence in improving their BP but no significant reduction in SBP.

Furthermore, mixed results were observed in studies with more than 1 primary aim (n=6, 21) [13,23,25,28,32,45]. For example, the study by Persell et al (2020) [23], which aimed to reduce SBP and increase self-confidence in BP control, reported patients’ increased self-confidence in their ability to control their BP but no significant reduction in SBP. On the other hand, the study by Schroeder et al (2020) [25], which aimed to reduce BP and improve medical adherence, reported no significant intervention impact on both aims.

In terms of study approach, all studies that implemented either an EHR-driven BPA (n=3, 10%) [37,47,48], clinical decision support (CDS; n=1, 3%) [45], case management (n=1, 3%) [49], and dashboard (n=3, 10%) [33] interventions reported a significant improvement in all their hypertension management outcomes. In comparison, most of the health coaching interventions (n=5, 17%) had no significant effect in their hypertension outcomes [13,23,25,35,40].

The outcomes also differed by the role of the EHR. Of the 22 (76%) studies that reported a significant change in at least 1 of the primary outcomes, 11 (50%) studies mentioned using the EHR for identification, intervention, and monitoring purposes [13,22,29,33,36-39,41,44,47]. Moreover, 2 (9%) studies utilized the EHR for identification and intervention [45,48], 1 (5%) study for identification and monitoring [30], 2 (9%) for intervention only [28,31], and 5 (23%) for identification only [24,26,45,48,49].

Finally, there were no noticeable differences in the relationship between study duration and results. Some study results were significant at a specific time point (n=4, 13%) [30,39,48,49] and others at different time points (n=3, 10%) [26,31,41]. To illustrate, the 9- and 18-month study by Hebert et al (2012) [26] showed a significant reduction in SBP at 9 months but not at 18 months. Similarly, the 6-, 12-, and 18-month study by Jackson et al (2012) [41] showed a significant reduction in SBP and DBP at 12 and 18 months but not at 6 months. On the other hand, the study by Kim et al (2014) [31] reported a significant reduction in SBP and DBP at 6 and 12 months but not at 18 months. In comparison, the study by Margolis et al (2013) significantly reduced both SBP and DBP at each of the time points of 6, 12, and 18 months, and the study by Persell et al (2018) [27] was not successful at reducing BP at 3, 6, or 12 months after the intervention.

## Discussion

### Principal Findings

Overall, this review identified several use cases for the integration of the EHR in supporting interventions in primary care to strengthen hypertension management among racial and ethnic minoritized groups. In the following sections, our findings regarding primary care settings, addressed populations, implemented interventions, and the role of the EHR will be discussed.

### Primary Care Settings

All interventions in the included studies were implemented in primary care. Interventions delivered in a primary care clinic (clinical-based setting) were most common in this review and accounted for 15 (52%) studies (about 28,000 patients). In contrast, home-based interventions were less common (n=13 (45%) but included a higher number of patients (about 44,000). Community-based interventions were the least common and accounted for only 1 (3%) study.

Primary care is the first and most important point of contact between the health system and the population it serves [51]. However, many patients face barriers that limit their access to health care services. This is particularly true for racial and ethnic minoritized groups [52,53]. Even when access-related factors such as the patient’s insurance status and income are taken into account, racial and ethnic minoritized groups are likely to receive poorer quality health care compared to non–minoritized groups [54]. The reasons for this situation are complex and may include poor access to transportation, limited health care resources, patient preferences, and differential treatment by providers [55]. Some evidence indicates that interventions aiming to strengthen primary care can help improve equity in health outcomes [16].

Regarding effectiveness, there is mounting evidence that primary care–based interventions can strengthen hypertension management [56-58]. For example, a review by Manalili et al (2021) [59] showed that person-centered quality improvement strategies (eg, case management, self-management promotion, patient/provider education) are effective at improving BP outcomes. Similar results were shown in this review. Unfortunately, only 1 (3%) community-based study could be included. Evidence suggests that community-based interventions can help to reduce health disparities [60].

### Addressed Populations

With over 50,000 patients spanning 26 (90%) studies, Black/African American patients represented the largest population in this review. In contrast, Hispanic/Latino patients were represented in 18 (62%) studies (about 13,000 patients), Asian American patients in 7 (24%) studies (about 6,000 patients), and American Indian/Alaskan Native patients in 2 (68%) studies (about 96 patients).

However, a closer look reveals large differences in the representation of the included populations regarding the design, settings, and approaches of the studies. For example, whereas Black/African American patients were included in studies with a wide variety of study designs, study designs for other populations, in particular American Indian/Alaskan Native participants, were more limited.

With respect to intervention settings, the majority of the patients included in home-based interventions were Black/African American and Hispanic/Latino. In contrast, the only population included in a community-based intervention was Asian American. Nevertheless, the largest differences between populations were related to the specific approach. Out of 11 approaches included in this review, American Indian/Alaskan Native patients were only addressed in 3 (27%) types of interventions (health coaching, telemedicine, and CDS), and Asian American patients were only addressed in 6 types of interventions (health coaching, BPA, DMP, home-based BP monitoring, CDS, and self-management). Neither American Indian/Alaskan Native patients nor Asian American patients were addressed in studies targeting medication management.

However, more unsettling is that Native Hawaiian/Pacific Islander patients were not addressed in any study. This is disconcerting as they often have a higher prevalence of hypertension compared to some other populations included in this review [9]. Unfortunately, this is not a new phenomenon. There is a longstanding discussion about the underrepresentation of racial and ethnic minoritized groups in clinical trials and health care–related research [61-63]. Besides Native Hawaiian/Pacific Islander patients, American Indian/Alaskan Native and Asian American patients are often underrepresented [64-66]. Underrepresentation is problematic, as those who face the greatest health challenges often receive the least benefit from advancing evidence because they are not adequately represented in research studies [67]. Future studies should address this gap to decrease disparities and improve equality.

### Interventions to Strengthen Hypertension Management

Regarding interventions, health coaching was the most common primary approach, but it was often included as a secondary component across the interventions. Given that health coaching is considered the standard of care for improving the chronic health conditions of minoritized groups in primary care [68] as well as reducing chronic health disparities [69], this is not surprising.

Health coaching interventions provide an opportunity to increase the reach, capacity, and utilization of health care services, especially in low-income and underserved communities where health care systems may be limited [70]. Racial and ethnic minoritized groups, unfortunately, are predominantly concentrated in these communities [52].

As shown in our review, health coaching can be integrated as part of a technological-based strategy for addressing racial and ethnic disparities in care. A 2012 systematic review analysis by Chin and colleagues [69] identified that information technology–assisted tools, including the use of interactive computerized education and counseling, culturally tailored programs, and skill-based training, were common health coaching and educational intervention strategies for reducing disparities in health care.

One major reason for the use of EHR-assisted health coaching tools for racial and ethnic minoritized groups may be due to the need to address patient-provider communication barriers, health literacy challenges, and health system or provider mistrust [71]. The use of these tools can help bridge gaps by increasing patient engagement through culturally tailored information sharing, ultimately improving patient-provider communication and the quality of health service delivery.

However, regarding effectiveness, the EHR-driven health coaching interventions in our review were largely unsuccessful in improving hypertension control and management. Only 3 (38%) of the 8 health coaching studies reported a significant improvement in hypertension control. Our findings conflict with the results of other systematic studies suggesting that health coaching interventions can significantly improve BP outcomes [68,72-75]. The observed conflict with our findings may be due to the EHR focus of our review or differences in population, intervention setting, study design, study duration, or how the health coaching components were implemented.

In contrast to health coaching interventions, all studies implementing BPA and CDS approaches significantly improved BP and hypertension outcomes in the participating racial and ethnic minoritized groups. Overall, BPA and CDS can be powerful tools for addressing disparities, provided that the decision aids in the systems are incorporating evidence-based and standardized guidelines. For example, Leewen [63] recommends linking CDS contents to local social services for recommended interventions, ensuring that providers with expertise in disadvantaged communities are included in CDS development and implementations, pilot testing CDS tools in real-world settings that include disadvantaged communities, and prioritizing clinical recommendations based on cost-effectiveness.

### Role of the EHR

All studies included in this review used the EHR in some capacity to identify patients, drive interventions, and monitor results. For identifying patients, the EHR was usually used to select specific patient groups based on health status (eg, with uncontrolled BP) or social demographics (eg, based on ethnicity) and to develop registries. Regarding driving interventions, the EHR was commonly utilized to trigger or alert processes and actions, alter the course of treatment or intervention, or both. In particular, in many studies, the EHR enabled tailoring interventions, including behavioral or educational massages and medication or treatment plans. It was also used to trigger context- or patient-specific events. As for monitoring results, the EHR was used, for example, to monitor BP changes, health care utilization, prescriptions, or the occurrence of adverse health events.

Considered separately, the described use cases are well established. However, the level of conceptualization as a unified strategy to improve hypertension management is low. Although research is rapidly expanding on how to use information and communication technology to support health and health care, often referred to as eHealth or digital health, there is a lack of clarity and consistency in the definition and use of the related terms [76].

This is especially true for data-driven approaches. Most existing concepts or definitions of data-driven approaches are broad. For example, one definition describes data-driven approaches as “technologies that work by collecting, using, and analyzing patient data to support the care of individuals…services, public health, or medical research and innovation” [19,77]. To the best of our knowledge, there is currently no detailed conceptualization of EHR-driven approaches.

However, besides the studies included in this review, many EHR-based approaches fit under this umbrella definition, such as EHR-driven phenotyping [78-80], workflows and decision support [81-83], and prediction-based interventions [84-86]. Whereas those approaches address a specific function, the differentiation between “identifying patients,” “driving interventions” and “monitoring results” is much broader and addresses the overall role of the EHR in supporting treatment and care. This more simplistic taxonomy may be helpful as a first step to gaining a better understanding of how to utilize the EHR to support hypertension management among racial and ethnic minoritized groups.

### Strengths and Limitations

To our knowledge, this is the first systematic review of EHR-driven interventions to improve hypertension management among racial and ethnic minoritized groups in primary care. We found that the EHR can serve multiple roles, ranging from clinical-level practices (eg, early identification of at-risk patient groups or patient-tailored treatment) to patient-level practices, such as self-care-based management of hypertension. Our findings provide critical insights for future research on improving hypertension health outcomes in racial and ethnic minoritized groups.

Our study also has limitations. First, we restricted our searches to English-language publications and studies conducted in the United States. These restrictions limited the number of available studies for review. Additionally, due to the focus on racial and ethnic minoritized groups in the United States, the overall findings may not be generalizable to minority populations in other countries. Second, we assessed all study designs as part of our inclusion criteria and were not restricted to just RCTs. Our review consisted of studies that also utilized CRT and non-RCT study designs. These studies were included due to our primary research aim, which was to identify and categorize evidence of EHR-driven approaches in primary care-level interventions. The synthesis of different study designs may have impacted our overall interpretations of study effectiveness by the relevant study characteristics. Third, we experienced difficulties in accurately categorizing the role of EHR. Some studies did not explicitly describe data utilization. We included these studies in the final analysis due to specific components of the intervention methodology, which pointed to EHR use. Fourth, it was challenging to accurately ascertain the effectiveness of some interventions. The reported study methods and results did not differ by duration. Some studies measured and reported the intervention impact at 6, 12, or 18 months after the study period, and others reported all 3 durations. The studies provided no rationale for the timelines used. The different follow-up periods could result in an overestimation or underestimation of the intervention’s effectiveness. These findings reflect the current state of EHR integration, including the limited research surrounding best practices for EHR-integrated interventions. Despite these limitations, we remain confident in the depth of our analysis, findings, and overall conclusions.

### Conclusions

This review identified several use cases for the integration of the EHR in supporting primary care interventions to strengthen hypertension management in racial and ethnic minoritized patients in the United States. Some clinical-based interventions implementing CDS and BPA applications showed promising results. However, more research is needed on community-based interventions, particularly those focusing on patients who are Asian American, American Indian/Alaskan Native, and Native Hawaiian/Pacific Islander. The developed taxonomy comprising “identifying patients,” “driving intervention,” and “monitoring results” to classify EHR-driven approaches can be a helpful tool to facilitate this.

## Appendix

Multimedia Appendix 1 PRISMA (Preferred Reporting Items for Systematic Reviews and Meta-analyses) checklist.

Multimedia Appendix 2 Randomized controlled trials (RCTs).

Multimedia Appendix 3 Cluster randomized trials (CRTs).

Multimedia Appendix 4 Non–randomized controlled trials (RCTs).

Multimedia Appendix 5 Clinical-based interventions.

Multimedia Appendix 6 Home-based interventions.

Multimedia Appendix 7 Community-based interventions.

## Abbreviations

BP: blood pressure
BPA: best practice alert
CCM: chronic care model
CDS: clinical decision support
CRT: cluster randomized trial
DBP: diastolic blood pressure
DMP: disease management program
EHR: electronic health record
PICO: Population, Intervention, Comparison, and Outcome
PRISMA: Preferred Reporting Items for Systematic Reviews and Meta-analyses
RCT: randomized controlled trial
RoB 2: Cochrane Risk-of-Bias version 2
ROBINS-I: Risk of Bias in Nonrandomized Studies
SBP: systolic blood pressure
