# Supplementary figures and images for: Electronic Health Record–Driven Approaches in Primary Care to Strengthen Hypertension Management Among Racial and Ethnic Minoritized Groups in the United States: Systematic Review
Source: J Med Internet Res. 2023 Sep 15;25:e42409. doi: 10.2196/42409 (PMC10541643; doi:10.2196/42409)

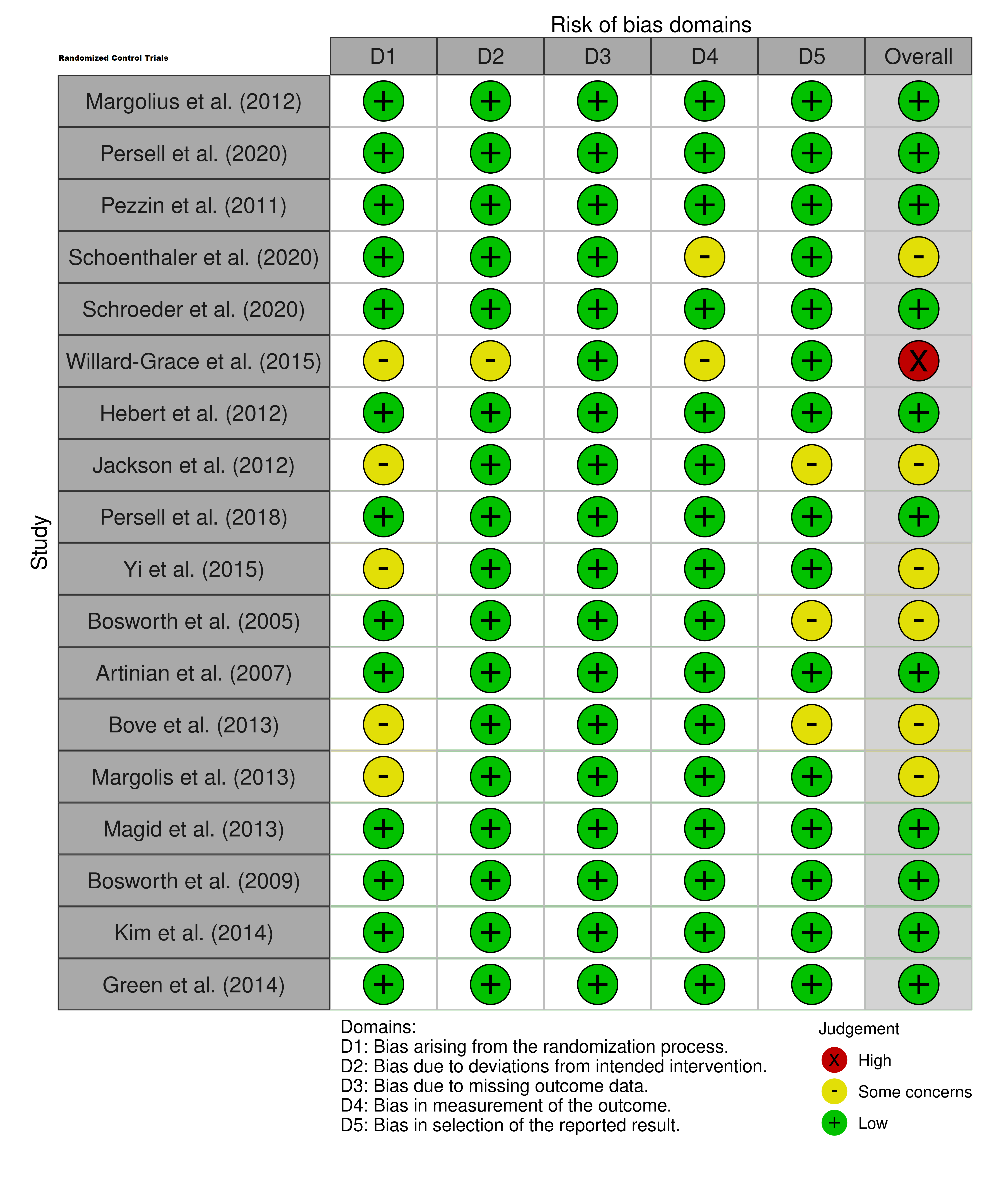

Supplement: Multimedia Appendix 2 [file jmir_v25i1e42409_app2.png]

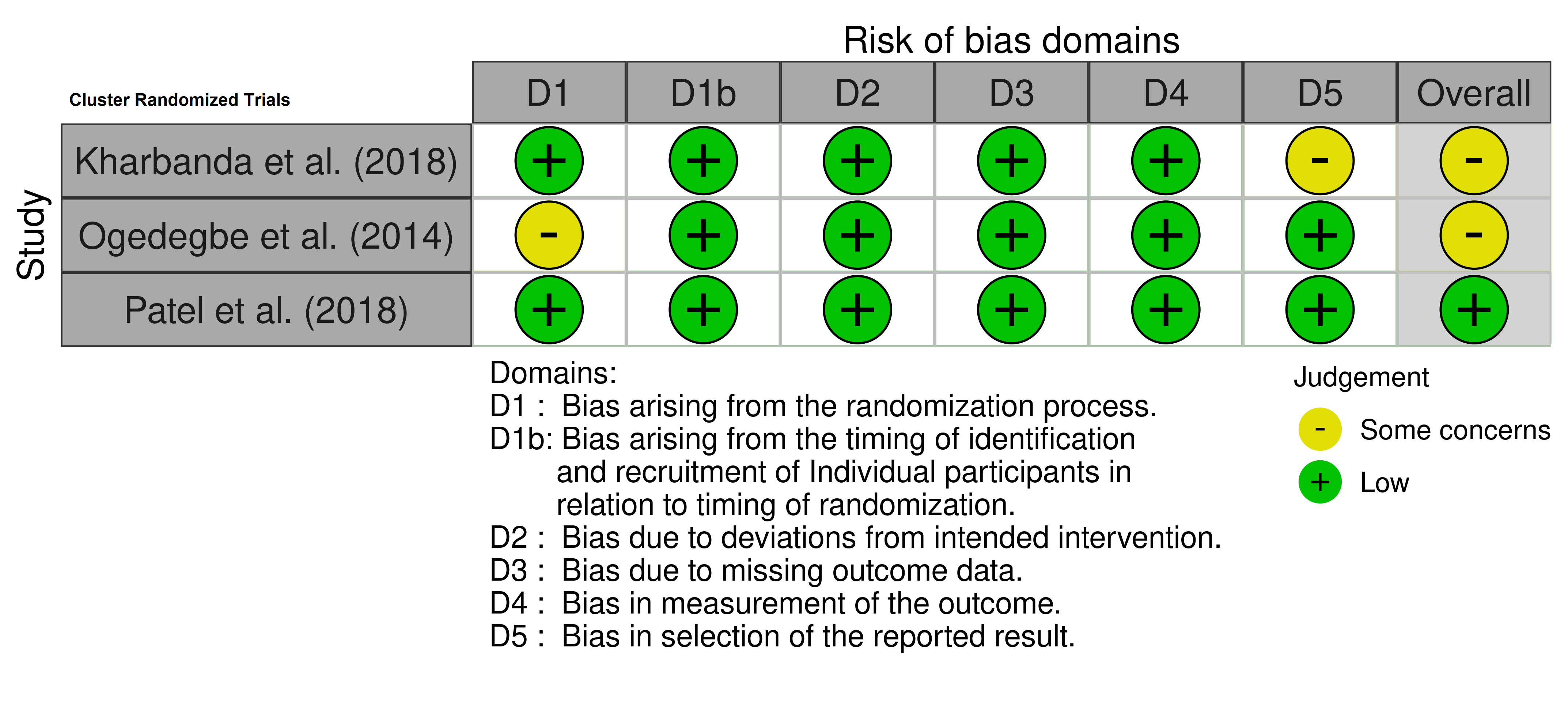

Supplement: Multimedia Appendix 3 [file jmir_v25i1e42409_app3.png]

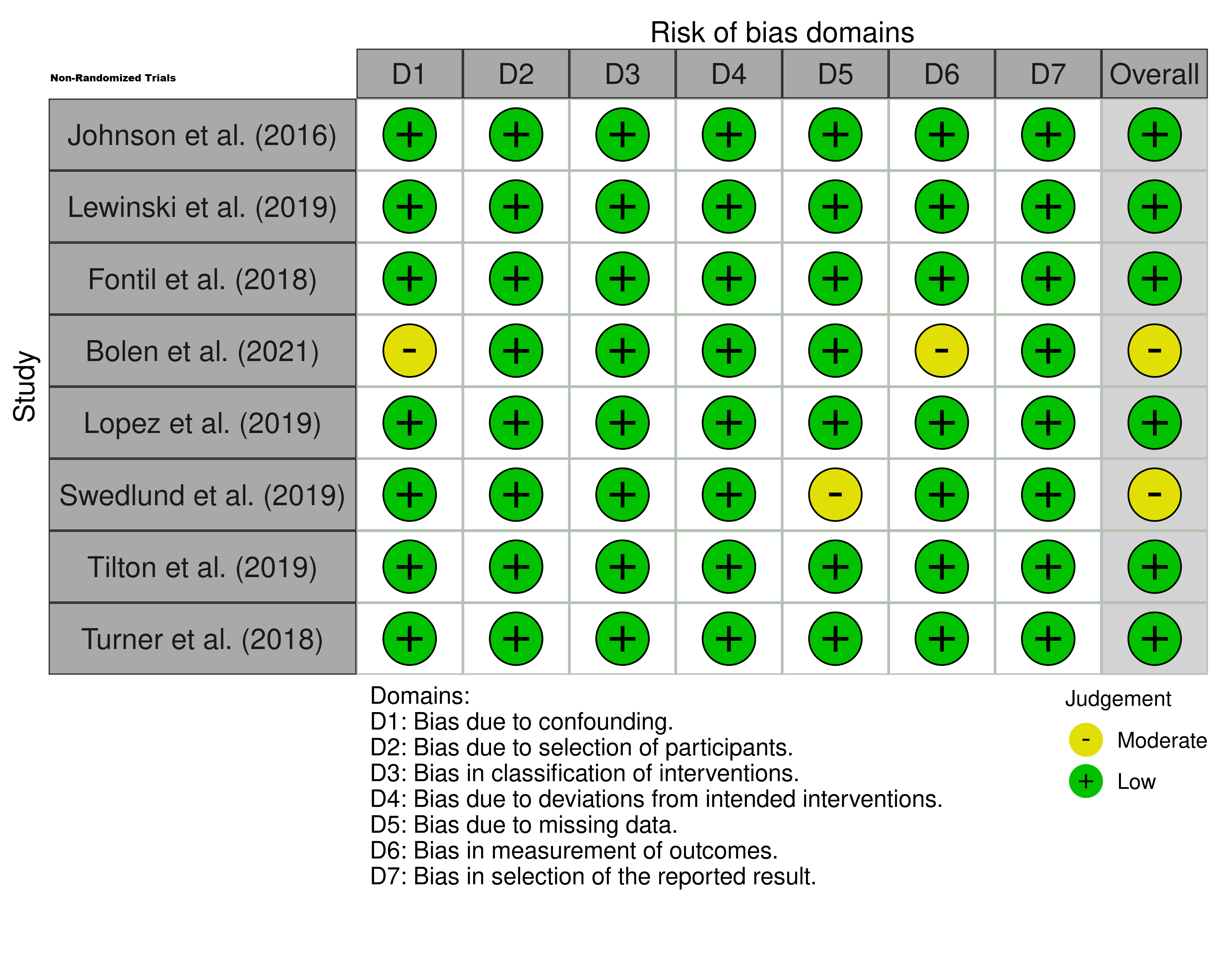

Supplement: Multimedia Appendix 4 [file jmir_v25i1e42409_app4.png]
