# Supplementary material for: Electronic Health Record–Driven Approaches in Primary Care to Strengthen Hypertension Management Among Racial and Ethnic Minoritized Groups in the United States: Systematic Review
Source: J Med Internet Res. 2023 Sep 15;25:e42409. doi: 10.2196/42409 (PMC10541643; doi:10.2196/42409)
Supplement: Multimedia Appendix 5 [file jmir_v25i1e42409_app5.docx]

**eTable 1:** Clinical-based Interventions

| **Author** | **Populations** | **Study Design** | **Aim** | **Intervention** | **Results** |
| --- | --- | --- | --- | --- | --- |
| Willard-Grace et al. (2015) | Asian (n=18) B/AA (n=84)  H/L (n=309) | Randomized controlled trial | - Improve self-management | Health coaching | - No significant increase in patients meeting SBP goals |
| Johnson et al. (2016) | B/AA (n=3) | Non-randomized feasibility study | - Improve Self-management | Health coaching | - 89% of patients maintained communication with health coach, 44% of patients reported BP monitoring |
| Lewinski et al. (2019) | B/AA (n=99),  H/L (n=2) | Pragmatic study | - SBP↓ | Health coaching | - No significant decrease in SBP |
| Schoenthaler et al. (2020) | H/L (n=119) | Randomized controlled trial | - Med. adherence↑ - BP↓ | Health coaching | - No significant increase in medication adherence, - Decreased SBP (p=0.02) but not DBP |
| Fontil et al. (2018) | Asian (n=5,506)  B/AA (n=3,597) H/L (n=3,530) | Pragmatic study | - BP control↑ | DMP | - Improved BP control rates (p<0.01) across all racial ethnic groups |
| Jackson et al. (2012) | B/AA (n=284) | Randomized controlled trial | - SBP↓ - DBP↓ | DMP | - Decreased SBP at 12 month (p=0.03) and 18 months (p=0.003), Decreased DBP at 18 months (p=0.01) |
| Hebert et al. (2012) | B/AA (n=246)  H/L (n=152) | Randomized controlled trial | - SBP↓ - DBP↓ | DMP | - No significant improvement in DBP - Decreased SBP at 9 months (p=0.018) |
| Lopez et al. (2019) | Asian (n=342)  B/AA (n=110)  H/L (n=54) | Quasi-experimental study | - BP control↑ | BPA, order-sets | - Improved BP control (RR: 1.09, 95% CI: 1.04-1.14), decreased SBP and DBP (p<0.05) |
| Swedlund et al. (2019) | BAA (n=1,504)  H/L (n=671) | Retrospective cohort study | - BP control↑ | BPA | - Improved BP control at follow-up (p<0.001) |
| Kharbanda et al. (2018) | Asian (n=128)  B/AA(n=231)  H/L (n=81) | Cluster randomized trial | - Diagnosis of elevated BP↑ - Follow-up↑ | CDS | - Increased diagnoses of elevated BP (p<0.001); - Increased follow-ups for BP (p=0.007) |
| Persell et al. (2018) | B/AA (n=692) | Randomized clinical trial | - Med. adherence↑ - BP↓ | Medication management | - No significant decrease in medication adherence - No significant decrease in BP |
| Tilton et al. (2019) | B/AA (n=224)  H/L(n=34) | Retrospective matched cohort study | - SBP↓ - DBP↓ | Medication management | - Decreased SBP (P=0.0160) and DBP (P=0.0136) at 6 months, decreased SBP (P=0.0018) at 12 months |
| Ogedegbe et al. (2014) | B/AA (n=1039) | Cluster randomized trial | - BP control↑ | CCM | - No significant difference in BP control rate between the intervention arms |
| Turner et al. (2018) | H/L (n=4,536) | Retrospective cohort study | - BP control↑ | CCM | - Increased rates of controlled BP (p=0.001) |
| Patel et al. (2018) | B/AA (n=4,774) | Cluster randomized trial | - Statin prescription↑ | Dashboard implementation | - Increased rates of physician-signed statin prescription orders (p=0.008) |

B/AA: Black/African American; AI/AN: American Indian/ Alaska Native; H/L: Hispanic/Latino; BP: Blood pressure; BPA: Best practice alerts; CCM: Chronic care model; CDS: Clinical decision support; DBP: Diastolic Blood pressure; DMP: Disease management program; HTN: Hypertension; SBP: Systolic Blood pressure;
