# Supplementary material for: Electronic Health Record–Driven Approaches in Primary Care to Strengthen Hypertension Management Among Racial and Ethnic Minoritized Groups in the United States: Systematic Review
Source: J Med Internet Res. 2023 Sep 15;25:e42409. doi: 10.2196/42409 (PMC10541643; doi:10.2196/42409)
Supplement: Multimedia Appendix 6 [file jmir_v25i1e42409_app6.docx]

**eTable 2:** Home-based Interventions

| **Author** | **Populations** | **Study Design** | **Aim** | **Approach** | **Results** |
| --- | --- | --- | --- | --- | --- |
| Margolius et al. (2012) | Asian (n=71)  B/AA (n=23)  H/L (n=94), | Randomized controlled trial | - BP control↑ | Health coaching | - Improved BP control (p<0.001) |
| Persell et al. (2020) | Asian (n=13), B/AA (n=103) | Randomized clinical trial | - SBP ↓ - Self-confidence in BP control↑ | Health coaching | - No significant decrease in SBP - Increased self-confidence in BP control (p<0.001) |
| Pezzin et al. (2011) | B/AA (n=845) | Randomized controlled trial | - BP control↑ | Health coaching | - Improved BP control (p=0.01) in patients with stage 2 uncontrolled HTN |
| Schroeder et al. (2020) | B/AA (n=14)  H/L (n=153)  AI/AN (n=74) | Randomized clinical trial | - BP↓ - Med. adherence↑ | Health coaching | - No significant decrease in BP, no impact on medication adherence |
| Bolen et al. (2021) | B/AA (n=37,359)  H/L (n=3,449) | QI study | - BP control↑ | BPA | - Improved BP control overall by 7.6% (95% CI 6.0-9.1) |
| Artinian et al. (2007) | B/AA (n=387) | Randomized control trial | - BP↓ | Telemedicine/ Virtual Visits | - Decreased SBP (p=0.04), no significant reduction in DBP (p=0.12) at 12-months |
| Bove et al. (2013) | B/AA (n=195)  H/L (n=7) | Randomized control trial | - BP control↑ | Telemedicine/ Virtual Visits | - No significant reduction in SBP and DBP at 6-months |
| Margolis et al. (2013) | B/AA (n=53)  H/L (n=10)  Other (n=22) | Randomized control trial | - BP control↑ | Telemedicine/ Virtual Visits | - Decreased SBP and DBP at 6-months (p<0.0001, P<0.0001), 12-months (p<0.0001, p<0.001), 18-months (p=0.004 & p=0.07) |
| Yi et al. (2015) | B/AA (n=218)  H/L (n=523) | Randomized control trial | - BP control↑ | BP Home monitoring | - No significant increase in BP control |
| Magid et al. (2013) | Asian (n=6)  B/AA (n=29)  H/L (n=26) | Randomized clinical trial | - BP control↑ | BP Home monitoring | - Decreased BP (P<0.01) at 6-months |
| Bosworth et al. (2005) | B/AA (n=236) | Randomized control trial | - Med. adherence↑ - Health behavior↑ | Case management | - Increased confidence with following HTN treatment protocols (p<0.007) |
| Bosworth et al. (2009) | B/AA (n=312) | Randomized control trial | - SBP↓ - DBP↓ - BP control↑ | Self-management | - Improved overall BP (p=0.012), SBP (p=0.010), DPB (p=0.009) at 24 months, similar at 12-months |
| Green et al. (2014) | Asian (n=3)  B/AA (n=5), | Randomized clinical trial | - SBP↓ - DBP↓ - Med. adherence↑ | Web-based team care | - No significant reduction in SBP and DBP, no significant improvement in medical adherence |

B/AA: Black/African American; AI/AN: American Indian/ Alaska Native; H/L: Hispanic/Latino; BP: Blood pressure; BPA: Best practice alerts; CCM: Chronic care model; CDS: Clinical decision support; DBP: Diastolic Blood pressure; DMP: Disease management program; HTN: Hypertension; SBP: Systolic Blood pressure
