# Supplementary material for: Electronic Health Record–Driven Approaches in Primary Care to Strengthen Hypertension Management Among Racial and Ethnic Minoritized Groups in the United States: Systematic Review
Source: J Med Internet Res. 2023 Sep 15;25:e42409. doi: 10.2196/42409 (PMC10541643; doi:10.2196/42409)
Supplement: Multimedia Appendix 7 [file jmir_v25i1e42409_app7.docx]

**eTable 3:** Community-based Interventions

| **Author** | **Populations** | **Study Design** | **Aim** | **Approach** | **Results** |
| --- | --- | --- | --- | --- | --- |
| Kim et al. 2014 | Asian (n=440) | Randomized control trial | - BP control↑ | Self-management | - Significant reduction in SBP, DBP at 6-months (p=0.001, p=0.021). 12-months (p=0.002, p=0.011) - Not significant at 18-months |

BP: Blood pressure; DBP: Diastolic Blood pressure; SBP: Systolic Blood pressure
